# Prevalence of nutritional risk in the non-demented hospitalised elderly: a cross-sectional study from Norway using stratified sampling

**DOI:** 10.1017/jns.2015.8

**Published:** 2015-05-06

**Authors:** Helene K. Eide, Jūratė Šaltytė Benth, Kjersti Sortland, Kristin Halvorsen, Kari Almendingen

**Affiliations:** 1 Division of Medicine, Akershus University Hospital and Institute of Clinical Medicine, University of Oslo, Lørenskog, Norway; 2 Department for Health, Nutrition and Management, Faculty of Health Sciences, Oslo and Akershus University College of Applied Sciences, Oslo, Norway; 3 Institute of Clinical Medicine, Campus Ahus, University of Oslo and HØKH, Research Centre, Akershus University Hospital, Lørenskog, Norway

**Keywords:** Nutritional risk, Elderly, Hospital practice, Cross-sectional studies, Stratified sampling, NRS2002, Nutritional Risk Screening 2002, S1, student 1, S2, student 2

## Abstract

There is a lack of accurate prevalence data on undernutrition and the risk of undernutrition among the hospitalised elderly in Europe and Norway. We aimed at estimating the prevalence of nutritional risk by using stratified sampling along with adequate power calculations. A cross-sectional study was carried out in the period 2011 to 2013 at a university hospital in Norway. Second-year nursing students in acute care clinical studies in twenty hospital wards screened non-demented elderly patients for nutritional risk, by employing the Nutritional Risk Screening 2002 (NRS2002) form. In total, 508 patients (48·8 % women and 51·2 % men) with a mean age of 79·6 (sd 6·4) years were screened by the students. Mean BMI was 24·9 (sd 4·9) kg/m^2^, and the patients had been hospitalised for on average 5·3 (sd 6·3) d. WHO's BMI cut-off values identified 6·5 % as underweight, 48·0 % of normal weight and 45·5 % as overweight. Patients nutritionally at risk had been in hospital longer and had lower average weight and BMI compared with those not at risk (all *P* < 0·001); no differences in mean age or sex were observed. The prevalence of nutritional risk was estimated to be 45·4 (95 % CI 41·7 %, 49·0) %, ranging between 20·0 and 65·0 % on different hospital wards. The present results show that the prevalence of nutritional risk among elderly patients without dementia is high, suggesting that a large proportion of the hospitalised elderly are in need of nutritional treatment.

Undernutrition and the risk of undernutrition constitutes a serious public health problem today and occurs frequently among the hospitalised elderly in developed countries^(^^1^^)^. However, the extent of the problem is not well described in the relevant literature and there is a lack of accurate prevalence data in Europe and Norway. Many of the studies conducted are based on small or narrowly defined hospital populations, or use inadequate statistical sampling methods when collecting data – all of which affect the prevalence estimates in an unfavourable way. Besides, different measurement methods are often employed as there is currently no clear consensus for a ‘gold standard’ method^(^^1^^,^^2^^)^. Estimates between 50 and 75 % are reported in a few Norwegian studies conducted in recent decades^(^^3^^–^^5^^)^. Prevalence estimates vary even more in a number of European studies^(^^6^^–^^13^^)^. The present study, therefore, aims to add to the body of quality prevalence data by providing prevalence estimates that meet strict methodological criteria.

Ageing results in physiological, psychological and social changes such as reduced lean body mass, impairment of senses like taste and smell, loneliness and cognitive impairment – all of which may contribute to the development of undernourishment^(^^14^^)^, again exacerbated by the presence of acute illness^(^^1^^,^^15^^)^. Moreover, most hospitalised elderly have chronic diseases and multiple diagnoses^(^^16^^)^, which in turn increase the risk of undernutrition^(^^1^^,^^15^^)^. If untreated, undernutrition can result in a variety of negative consequences and is associated with higher morbidity and mortality rates, more frequent complications, and longer hospital stays^(^^1^^,^^15^^)^.

In recent years, international and national guidelines have been published in Europe^(^^17^^,^^18^^)^ and in Norway^(^^19^^)^ to prevent and treat undernutrition in healthcare institutions effectively. The guidelines recommend that all patients in hospital care must be screened for nutritional risk on admission so that affected patients are identified^(^^17^^–^^19^^)^, a recommendation mandatory by law in Norway^(^^19^^)^. The goal of nutritional risk screening is to evaluate whether nutritional treatment is likely to influence the patients' outcome^(^^17^^)^. Such a screening aims to identify already undernourished patients and patients at risk^(^^17^^,^^19^^)^. A variety of nutritional risk screening tools have been developed and published for use in the hospital setting^(^^20^^,^^21^^)^, most of them based on recent weight loss, food intake and BMI^(^^2^^,^^17^^)^. Disease severity is also accounted for in some of the tools since stress metabolism may increase the patients' nutritional needs^(^^2^^,^^17^^)^.

The increasing number of elderly individuals contributes to substantial challenges for the healthcare sector^(^^16^^)^. Prevention and treatment of undernourishment in the elderly are thus of great importance and may yield both health- related and financial benefits. Awareness of incidence and prevalence estimates is central in highlighting the problem of undernourishment in the elderly, and is important for allocating healthcare resources. To our knowledge, no adequately designed prevalence study on undernutrition and the risk of undernutrition has previously been conducted among the hospitalised elderly in Norway. Since Norway represents a typical modern Western society, such a study would provide important insights into the problem of undernourishment among the hospitalised elderly in Scandinavia as well as in Western Europe.

The present study is specifically targeted at estimating the prevalence of nutritional risk among elderly hospitalised patients. A stratified sampling technique reducing sampling error was utilised in data collection to improve the representativeness of the sample. Adequate power calculations based on rather strong assumptions were performed *a priori* to assure an accurate estimate of the prevalence.

## Methods

### Study design

A cross-sectional study was designed and carried out at one university hospital in Norway. The university hospital operates as both a local and regional hospital, thereby offering locally based specialist healthcare services as well as more specialised services. The hospital covers about 10 % of the Norwegian population, providing healthcare services for approximately half a million individuals living in urban and rural municipalities. The patient population is heterogeneous with respect to ethnicity and socio-economic factors, and could be considered representative of Norwegian society.

The study was developed by a collegium at a nursing bachelor education programme in a multidisciplinary collaboration with representatives from the university hospital and other experts. All second-year nursing students at the university college in question who were undergoing their acute and clinical care practice studies at the university hospital were instructed to screen elderly patients for nutritional risk. The bachelor nursing education programme has a particular focus on nutrition, and the screening was an important part of the students' clinical training and education. To meet the substantial challenges related to undernourishment in the hospital setting, it is vital that nursing students receive proper education and training in nutritional risk screening. Involving students in research activities is also of importance for the university college offering the study programme to strengthen evidence-based practice.

**Fig. 1.**
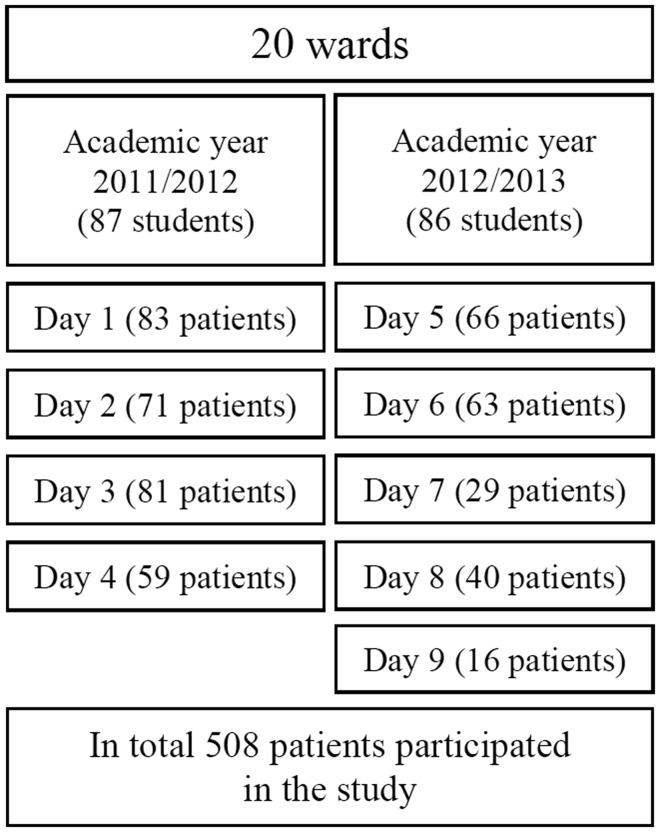
Study design. In total 508 hospitalised elderly (≥70 years) patients participated in the study. All second-year nursing students who were undergoing their acute and clinical care practice studies conducted nutritional risk screening on twenty hospital wards. Nine nutritional screening days were conducted in the academic years 2011/2012 and 2012/2013.

Totally, fourteen of sixteen medical and surgical somatic wards at the university hospital were included in the study. Additionally, one rehabilitation ward, one specialised short-term unit, one emergency medicine ward and one cardiac monitoring ward were included. Two wards were each divided into two sub-wards due to differences in the patients' diagnoses. Naturally, it was reasonable to assume that each of the twenty wards (Fig. 1) represented a homogeneous subgroup of the patient population. Data were therefore collected by using stratified sampling^(^^22^^)^, with the wards defined as strata. Stratified sampling is known to be the most representative of a population in the sense of minimised sampling error. A statistician was responsible for the statistical sampling design.

### Selection of participants

Nine nutritional screening days were conducted in the academic years 2011/2012 and 2012/2013 (Fig. 1). The screening days were Tuesdays, Wednesdays and Thursdays to ensure a steady coverage of patients, as most patients are admitted on Mondays and discharged on Fridays. The data were collected by 173 students. All the students were informed about the study and introduced to the screening form by a clinical dietitian at the start of the academic year. Shortly before each screening day, clinical supervisors (lecturers and professors) from the university college met the students in small groups to go through the questionnaire and the screening form carefully. A research and development nurse at the university hospital was employed by project funding to ensure better communication with the wards and to inform the ward staff about the screening.

All elderly (≥70 years) patients admitted on the included wards at 08.00 hours on the screening days were asked to participate. Eligible patients were selected by the students in cooperation with the ward nursing staff. Terminal patients, i.e. patients assumed short-lived (less than 1 month) and patients diagnosed with dementia were excluded. In addition, patients experiencing language difficulties, being scheduled for operations/examinations or unfit to participate were also excluded.

On the screening days the students filled in the questionnaire for each patient, including questions about age, sex, length of hospital stay, weight, height, BMI and nutritional risk. The students measured weight and height whenever possible, and screened the patients for nutritional risk. A specially prepared manual instructed the students on how to fill in the questionnaire and use the screening form properly. The students usually collected the data in pairs, making it possible for them to verify each other's work. Two individuals central to the research project were available to the students at the hospital on all screening days.

### Data collection

#### Anthropometric measurements

Weight was measured without shoes and outer clothes in either a standing or sitting position to the nearest 0·1 kg with the weight apparatus available on the different wards, following usual hospital practice. Height was measured to the nearest 1 cm with a non-elastic measuring tape either in a standing position against a wall without shoes or alternatively with the half arm-span method if the patients had problems standing^(^^23^^)^, a reliable substitute for standing height for the elderly^(^^23^^,^^24^^)^. BMI was calculated as weight (kg) divided by the square of height (m). The age-independent cut-off values presented by the WHO^(^^25^^)^ were used when categorising patients' BMI.

#### Assessment of nutritional risk

The translated version^(^^19^^)^ of the validated^(^^26^^)^ Nutritional Risk Screening 2002 (NRS2002) form from 2009 was used to identify patients nutritionally at risk. The screening form is recommended by the European Society for Clinical Nutrition and Metabolism (ESPEN)^(^^17^^)^ and the Norwegian Directorate of Health^(^^19^^)^ for use in the hospital setting. The NRS2002 aims to detect patients who will benefit from nutritional treatment due to undernutrition and/or increased nutritional needs resulting from disease^(^^26^^)^. The screening form included an initial screening and a final screening (Appendix 1). The final screening was conducted if the answer was ‘yes’ to any one of the four questions in the initial screening. Patients with a total score of three or more were classified as nutritionally at risk. All scorings of nutritional risk were checked by a clinical dietitian shortly after each screening day.

### Pilot and inter-rater agreement studies

A pilot study involving 290 elderly patients and ninety-six nursing students was performed during the autumn of 2010 and the spring of 2011 at the university hospital to test the use of a nutritional risk screening form, as well as the additional questionnaire on the patients' demographic characteristics. The questionnaire was revised after the pilot study. The pilot study also confirmed that the bachelor nursing education programme had an infrastructure that enabled the collection of data.

As a large number of students was involved in data collection for the present study, the data quality might be questioned. An inter-rater agreement study on age, weight and height was therefore carried out. Two nursing students (students 1 and 2; S1 and S2) familiar with the ordinary screening study, but not a part of it, were trained to collect data for the agreement study. On the third and fourth screening days, shortly after the ordinary screening was completed, S1 and S2 independently of each other screened repeateadly thirty patients on seven wards. Data collected from S1 and S2 were later merged with the results of the ordinary screening for further analysis.

### Sample size

After a literature review and discussions with experts in the field, the proportion of elderly nutritionally at risk was assumed to be 30 %. According to the standard statistical power calculations, a total of 165 patients were needed to detect this large proportion with a 95 % CI of 10 % or less. To account for a possible clustering effect within wards, an intra-class correlation coefficient of 0·3 was assumed. The minimum number of patients required in the study to detect a prevalence of 30 % nutritionally at risk with a 95 % degree of confidence with a true population estimate between 25 and 35 % was then estimated to be 522. Subsequently, on each ward the number of elderly patients proportional to the ward size was consecutively included in the sample. The size of ward was defined as the daily average number of elderly patients based on the records from the last 6 months provided by the hospital's analysis department. Sampling stopped on each ward when the intended number of patients was reached.

### Data analysis

Demographic and clinical characteristics were described as mean values and standard deviations or as frequencies and percentages, as appropriate. Patient characteristics between those nutritionally at risk and not at risk were compared by a *t* test for independent samples for continuous variables and Fisher's exact test or χ^2^ test for categorical variables.

The prevalence of nutritional risk was estimated as suggested by Cochran^(^^22^^)^ in the following way: a proportion of patients nutritionally at risk in each stratum (ward), *p*_*h*_, was estimated first; here *h* = 1,2, …, 20 is the ward indicator. Then weights *W*_*h*_ were defined as the ratio of a ward size *N*_*h*_ to the total, defined as sum of all *N*_*h*_, i.e. 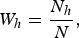 where *N* = ∑_*h*_ *N*_*h*_. Then the weighted prevalence was calculated as p = ∑_*h*_ *W*_*h*_*p*_*h*_. The variance of estimated prevalence was then defined as 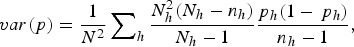 where *n*_*h*_ is the number of patients sampled in ward *h*.

Agreement in age, weight and height between the three students (S1, S2 and nursing students performing ordinary screening) was assessed by Bland–Altman analysis, where 95 % limits of agreement were constructed. The 95 % limits of agreement define an interval in which 95 % of differences between two scoring populations would lie. The acceptable limits were set *a priori* to ±1 year in age, ±2 kg in weight and ±3 cm in height. Bias, defined as the mean difference between measurements of two students, was assessed by one-sample *t* test.

The statistical program IBM SPSS Statistics version 20 for Windows was used for statistical analysis. *P* values below 0·05 were considered statistically significant. All tests were two-sided. Anonymous data files were analysed by a statistician.

### Ethics

The present study was conducted according to the guidelines laid down in the Declaration of Helsinki and procedures involving human patients were approved by the university hospital's Internal Privacy Commission. Verbal informed consent was obtained from all patients. Verbal consent was witnessed and formally recorded. As the screening data were anonymous, the study was exempted from review by the Regional Committees for Medical and Health Research Ethics (reference no. 2011/2088 A). The researchers received anonymously completed questionnaires and screening forms from the students and never met the patients. The ClinicalTrials.gov ID is NCT01977950 (http://www.clinicaltrials.gov).

## Results

### Participation

All elderly patients were approached on the nutritional screening days (Fig. 1). Only approximate information on participation status was known due to some students' incomplete reporting on seven wards. Of 1059 patients with known participation status, 145 patients (14 %) declined participation, while 390 (37 %) were excluded according to predefined criteria. In total, 508 patients (49 %) participated on the nine screening days. As a consecutive inclusion of patients was performed, a somewhat low participation rate does not affect the data quality. As estimated by the intra-class correlation coefficient, the cluster effect in the data was only 5·4 %, which is considerably lower than the 30 % assumed in power calculations. Consequently, a slightly lower sample size than expected (*n* 522) could not influence the precision of the prevalence estimate.

### Patient characteristics

Patient characteristics are outlined in Table 1. Of a total of 508 patients in the sample, 201 (39·6 %) were nutritionally at risk, 252 (49·6 %) were not nutritionally at risk, while nutritional risk was unknown in fifty-five cases (10·8 %). Reasons for unknown nutritional risk were missing data on weight for twelve of the patients (21·8 %), while eleven (20 %) could not recall previous weight. For the remaining thirty-two patients (58·2 %), the students had not filled out the screening form correctly. There were no statistically significant differences in mean age and sex between patients nutritionally at risk and patients not nutritionally at risk (Table 1). Notably, patients nutritionally at risk had been hospitalised for longer on the day the measurements were taken and had lower average weight and BMI compared with the patients not at risk (Table 1); all differences were statistically significant (*P* < 0·001). WHO BMI cut-off values^(^^27^^)^ identified 6·5 % as underweight, 48·0 % as of normal weight and 45·5 % as overweight.

**Table 1.** Patient characteristics (Mean values and standard deviations, or numbers of subjects and percentages)

	Total sample		Nutritionally at risk		Nutritionally not at risk		
	Mean	sd	Mean	sd	Mean	sd	*P*
Subjects							–
*n*	508		201		252		
%			39·6		49·6		
Sex (*n*)	502		199		249		0·506*
Men							
*n*	257		98		131		
%	51·2		42·8		57·2		
Women							
*n*	245		101		118		
%	48·8		46·1		53·9		
Age (years) (*n*)	505		201		250		
	79·6	6·4	80·1	6·1	79·4	6·4	0·262†
Length of stay (d) (*n*)	498		198		247		
	5·3	6·3	6·0	7·8	4·6	4·9	0·023†
Body weight (kg) (*n*)	492		200		249		
	71·3	16·5	63·8	15·7	76·6	15·1	<0·001†
BMI (kg/m^2^) (*n*)	492		199		250		
	24·9	4·9	22·4	4·6	26·7	4·4	<0·001†
BMI (WHO categories)^(^^25^^)^							<0·001‡
Underweight: ≤18·49 kg/m^2^							
*n*	32		32		0		
%	6·5		100		0		
Normal weight: 18·5–24·99 kg/m^2^							
*n*	236		115		103		
%	48·0		52·8		47·2		
Overweight: ≥25 kg/m^2^							
*n*	224		52		147		
%	45·5		26·1		73·9		

* Fisher's exact test.

† *t* Test for independent samples.

‡ χ^2^ test.

### Inter-rater agreement study

Descriptive analysis did not show any considerable differences in mean age, weight and height (Table 2). Consequently, there was no significant bias between pairs of students. Differences between S1 and S2 were marginal. Deviations between the nursing students performing ordinary screening and S1 or S2 were somewhat larger. The 95 % limits of agreement were slightly wider than the prespecified acceptable limits. These deviations, however, were caused by only a few values, as identified by assessing the Bland–Altman plots.

**Table 2.** Descriptive statistics of age, weight and height collected by nursing students (performing ordinary screening), student 1 (S1) and student 2 (S2), and bias between students (including *P* values for one-sample *t* tests) and 95 % limits of agreement (LoA)

	Age (years)	Weight (kg)	Height (m)
Nursing students			
*n*	30	29	30
Mean	77·90	69·97	1·68
sd	5·20	15·07	0·12
S1			
*n*	30	29	30
Mean	77·83	70·89	1·66
sd	5·38	15·35	0·13
S2			
*n*	30	29	30
Mean	77·83	70·89	1·66
sd	5·38	15·34	0·13
Nursing students *v.* S1			
Bias	0·07	–0·92	0·03
*P*	0·961	0·818	0·415
95 % LoA	–1·71, 1·85	–3·56, 1·71	–0·17, 0·23
Nursing students *v.* S2			
Bias	0·07	–0·92	0·03
*P*	0·961	0·818	0·437
95 % LoA	–1·71, 1·85	–3·56, 1·71	–0·14, 0·19
S1 *v.* S2			
Bias	0·00	0·003	–0·001
*P*	1·00	0·999	0·976
95 % LoA	N/A	–0·17, 0·17	–0·09, 0·09

N/A, not applicable.

### Prevalence of nutritional risk

The prevalence of nutritional risk was calculated based on 453 patients (89·2 % of the total sample) where the nutritional risk was available. The prevalence was estimated to be 45·4 (95 % CI 41·7, 49·0 %) (Table 3). Detailed estimates of the number of patients nutritionally at risk on each ward are presented in Table 3. The prevalence rates ranged between 20·0 and 65·0 % on different wards.

**Table 3.** Total prevalence estimate and proportions of patients nutritionally at risk on each ward (Numbers of subjects and percentages)

	Total sample		Nutritionally at risk		Unknown	
Ward	*n*	%	*n*	%	*n*	%
Orthopaedics 1	40	7·9	8	20·0	8	20·0
Ear–Nose–Throat/Gynaecology	15	3·0	7	46·7	2	13·3
Vascular/Thorax	18	3·5	10	55·6	2	11·1
Gastro Surgery lower	26	5·1	13	50·0	2	7·7
Gastro Surgery upper	18	3·5	9	50·0	0	
Urology	22	4·3	13	59·1	0	
Orthopaedics 2	46	9·1	19	41·3	9	19·6
Emergency Medicine	23	4·5	7	30·4	1	4·3
Neurology + Endocrinology	28	5·5	9	32·1	1	3·6
Infectious Medicine 1	37	7·3	14	37·8	8	21·6
Neurology/Stroke	14	2·8	4	28·6	2	14·3
Infectious Medicine 2	13	2·6	4	30·8	0	
Rehabilitation Neurology	36	7·1	10	27·8	6	16·7
Renal Medicine	15	3·0	7	46·7	1	6·7
Heart Medicine	30	5·9	8	26·7	3	10·0
Lung Medicine	50	9·8	30	60·0	4	8·0
Cardiology Medicine	37	7·3	9	24·3	2	5·4
Cardiac Monitoring	6	1·2	1	16·7	2	33·3
Haematology	14	2·8	6	42·9	1	7·1
Specialised Short-Term Unit	20	3·9	13	65·0	1	5·0
Total prevalence estimate (%)	45·4					
95 % CI (%)	41·7, 49·0					

## Discussion

The present results demonstrate that undernourishment is a serious public health problem among the hospitalised elderly in a modern Western society. For the total sample, the estimated prevalence of nutritional risk was as high as 45 %, suggesting that nearly half of the elderly patients without dementia were in a need of appropriate nutritional treatment. The findings suggest that much can be done to improve the nutritional status of the hospitalised elderly. Defining ways to prevent and treat this condition effectively in the hospital setting should therefore be given immediate high priority. This is the first prevalence study on this scale conducted among the hospitalised elderly in Norway.

The major strengths of the present study are its proper statistical sampling design and the adequate power calculations that were carried out before the study. The differences in the patients' diagnoses on different wards, comprising relatively homogeneous units, make the stratified sampling a preferred technique in the hospital population. This sampling technique ensures sufficient representation of each ward, which might be difficult to achieve with simple random sampling. In addition, it tends to produce more precise estimates of population parameters as compared with simple random sampling, since the variances of the entire sample are based on the variances within each stratum^(^^28^^)^. Even though other studies with large sample sizes have produced prevalence estimates with high precision^(^^7^^,^^8^^,^^10^^–^^12^^)^, they have either sampled from certain types of wards or by consecutively including all admitted patients. Consequently some wards may have been under- or overrepresented, making it unclear if the numbers are representative. Further, due to possible similarities in patient characteristics within the same ward, the presence of a cluster effect within each ward was assumed in the power calculations in the present study. Power calculations taking into account such a cluster effect correctly result in sample sizes larger than those of standard power calculations, assuring an adequate number of patients in the study. As estimated by the intra-class correlation coefficient, the cluster effect in our data was considerably lower than that assumed in power calculations. Thus, even though somewhat smaller than planned, the sample of the present study can be considered sufficient for a reliable prevalence estimate.

The study sample comprised nearly all somatic medical and surgical wards at the university hospital, in addition to four associated wards. The hospital offers locally based specialist healthcare services as well as services that are more specialised. In this way, the sample covers a heterogeneous population of elderly hospitalised patients with a large variety of potential diagnoses. Moreover, the heterogeneity of the patient population makes it comparable with Norwegian society as a whole. Unfortunately, for ethical and practical reasons it was not possible to include the patients from the psychiatric division and patients diagnosed with dementia in the present study. The estimated prevalence therefore cannot be generalised to the entire elderly population at the university hospital. Generalisations from cross-sectional studies are always challenging. The results, however, clearly show the extent of the problem of undernutrition and the risk of undernutrition among the hospitalised elderly in Norway today, although the estimated prevalence would probably have been even higher if elderly patients diagnosed with dementia had been included^(^^14^^)^. The present results are strengthened by the fact that similar figures have been shown in Europe^(^^13^^)^.

The hospital ward composition may make an impact on the total prevalence estimate, as our data indicate that the proportion of patients nutritionally at risk varied in wards. It has been argued that large hospitals tend to differ from other hospitals in terms of ward composition by providing more specialised care, which could affect the case mix of the studied population and further effect the prevalence estimate^(^^29^^)^. However, by providing more specialised care in addition to locally based specialist healthcare services, large hospitals usually handle a wider variety of potential diagnoses, and sampling from large hospitals will therefore ensure more representative data.

The results from other Norwegian^(^^3^^–^^5^^)^ and European studies^(^^6^^–^^13^^)^ reporting the prevalence of undernutrition and the risk of undernutrition among the hospitalised elderly have shown variable prevalence rates. This is presumably due to methodological differences and weaknesses, and the results are often not representative of the studied population and/or can seldom be generalised to a larger part of the elderly population at the hospital studied. Different measurement methods, such as screening tools and BMI cut-offs, are also often employed, which makes it challenging and even impossible to compare the results. Three studies that have employed the NRS2002 to identify nutritional risk have reported either lower (22–28 %)^(^^12^^)^, higher (54 %)^(^^6^^)^ or similar (42 %)^(^^13^^)^ rates compared with the present results. However, the published lower rate only reflects nutritional risk on hospital admission^(^^12^^)^. As nutritional status often deteriorates during hospital stays^(^^30^^)^ and undernourished patients in general are hospitalised longer^(^^1^^,^^15^^)^, rates on admission will usually be lower than estimates covering the entire hospitalised population. Moreover, none of the three studies used proper statistical sampling methods for estimating prevalence rates^(^^6^^,^^12^^,^^13^^)^, and only medical wards were included in the samples of the studies reporting either lower or higher rates^(^^6^^,^^12^^)^. The studies that report much higher prevalence rates compared with that in the present study have often used the screening form Mini Nutritional Assessment Tool (MNA)^(^^3^^,^^6^^–^^9^^,^^11^^)^, which has been shown to identify more patients nutritionally at risk compared with the NRS2002^(^^6^^,^^31^^,^^32^^)^. The MNA is specifically developed and recommended for use on elderly patients^(^^17^^,^^33^^)^. However, being of old age is also taken into account in the NRS2002^(^^26^^)^. The NRS2002 may also be a more appropriate screening form for use with the acutely diseased elderly since the MNA does not consider the effect of stress metabolism on nutritional needs^(^^6^^,^^32^^,^^34^^,^^35^^)^. Furthermore, as the screening was part of the students' clinical training and education in the present study, it was important to choose a screening tool commonly used in the hospital setting in Norway, recommended by the Norwegian Directorate of Health.

We observed no age difference between patients nutritionally at risk and patients not at risk. This was somewhat surprising since advanced age is a known risk factor of undernourishment^(^^14^^)^. On the other hand, this could be just an effect of the inclusion criteria (age ≥70 years), since younger patients were not included in the present study sample, compared with other studies that have found an effect of age on undernourishment^(^^10^^,^^12^^,^^13^^)^. In the present study we did not control for other patient characteristics, for example, multimorbidity, and the effect of age might have been dominated by other factors.

A limitation of the present study is that nutritional risk was unknown in 11 % of the sample, most often due to incomplete screening forms. We observed no systematic incomplete data; hence the impact of missing data on the prevalence estimate in the present study is considered to be minor. Another reason for missing data on nutritional risk was that a few patients could not recall previous weight, and the question of whether screening forms that require data on recent weight loss, like NRS2002, are suitable for the entire elderly hospitalised population, can be raised. However, as we excluded patients diagnosed with dementia, this information was lacking for only a few patients in the present study. On some wards a greater number of patients were excluded than on others. There is a risk that attitudes among the ward nursing staff may have led to unnecessary exclusion of patients found unfit to participate, which could have an impact on the estimate. Unfortunately, there was no detailed information on patient exclusion in the present study, and future studies should note the importance of obtaining such information.

The multidisciplinary collaboration was essential for carrying out the present study. Using students in this clinical study enabled a collection of a large dataset using limited resources. Moreover, the students gained insight into how a large multidisciplinary research study is planned and carried out, and they acquired important research-based professional knowledge and training in nutritional risk screening. The screening may also have induced increased competence among hospital ward staff. On the other hand, a large number of students involved in data collection might be seen as a shortcoming of the study. We can also assume that the students had limited research experience. However, the inter-rater agreement study exhibited an acceptable quality of the screening data. Moreover, individuals central to the planning and conducting of the present study were experienced in using students for the collection of research data^(^^36^^)^. The students also recieved supervision before each nutritional screening day, to secure the data collection.

In conclusion, the prevalence of nutritional risk among elderly without dementia was high, suggesting that a large proportion of hospitalised elderly patients are in need of nutritional treatment. The present study demonstrates how a close multidisciplinary collaboration between a university hospital and a nursing bachelor education programme can facilitate the conducting of a larger research study by involving students in research activities.

## Appendix 1. The Norwegian version of Nutritional Risk Screening 2002 (NRS2002) from 2009 (in English)^(^^19^^)^

**Table:**

Initial screening:				Yes	No
1. Is BMI <20·5 kg/m^2^?					
2. Has the patient lost weight within the last few weeks?					
3. Has the patient had a reduced dietary intake in the last weeks?					
4. Is the patient severely ill?					
Yes: If the answer is ‘Yes’ to any question, the final screening is performed					
No: If the answer is ‘No’ to all questions, the patient is rescreened at weekly intervals. If the patient is scheduled for a major operation, a preventive nutritional care plan is considered to avoid the associated risk status					
Final screening:					
Score	Nutritional status	Score	Severity of disease		
0	Normal nutritional status	0	Not ill		
1	Weight loss 5–10 % in the last 3 months and/or dietary intake of 50–75 % of requirement for a week or more	1	A patient with a chronic disease or a patient who has undergone minor surgery. Studies have been conducted on patients with liver cirrhosis, kidney failure, chronic lung disease and cancer, and on patients with collum femoris fracture, after cholecystectomy, and laparoscopic surgery		
2	BMI 18·5–20·5 kg/m^2^ and/or recent weight loss 10–15 % in the last 3 months and/or dietary intake of 25–50 % of requirement for a week or more	2	A patient with significantly reduced general condition due to illness. Studies have been conducted on patients with severe pneumonia, inflammatory bowel disease with fever, acute renal failure, major surgery such as colectomy and gastrectomy, ileus, anastomosis leakage and repetitive operations		
3	BMI ≤18·5 kg/m^2^ and/or recent weight loss >15 % in the last 3 months and/or dietary intake of 0–25 % of requirement for a week or more	3	A patient is seriously ill. Studies have been conducted on patients with large apoplexy, severe sepsis, intensive care unit patients (APACHE >10), bone marrow transplants, major head injuries, burns >40 % and severe acute pancreatitis		
Explanation of the final screening:					
The patient scores from 0 to 3 for nutritional status					
The patient scores from 0 to 3 for disease severity					
A score of 1 is added for patients older than 70 years					
If total score is ≥3, the patient is nutritionally at risk, and nutritional treatment must be initiated					
If total score is <3, the patient is not nutritionally at risk. Screening must be repeated after 1 week					

APACHE, Acute Physiology and Chronic Health Evaluation.
